# Supplementary material for: Treatment outcomes of diabetic ketoacidosis among diabetes patients in Ethiopia. Hospital-based study
Source: PLoS One. 2022 Apr 5;17(4):e0264626. doi: 10.1371/journal.pone.0264626 (PMC8982884; doi:10.1371/journal.pone.0264626)
Supplement: Attachment — Submitted filename: Response to reviewers.docx [file pone.0264626.s002.docx]

**Authors reply to reviewers’ comments**

PONE-D-18-29190: TREATMENT OUTCOME OF DIABETIC KETOACIDOSIS AMONG PATIENTS ATENDING GENERAL HOSPITAL IN NORTH-WEST ETHIOPIA: HOSPITAL BASED STUDY

3. **Please change your reference to "p=0.000" to "p<0.001" or as similarly appropriate, as p values cannot equal zero.**

Our reference to significant values will be "p<0.001".
5. **Review Comments to the Author**
Reviewer #1: The manuscript reports outcome of a large cohort of patients admitted for DKA in a Ethiopian hospital. These data may be of interest for the definition of outcomes of DKA in different clinical settings. However, the manuscript in the present form shows several major flaws which need to be amended.
1. **Only patients with complete records were included in the analysis. Please specify the minimum data set for inclusion, and the number of patients discarded for incomplete data. A sensitivity analysis on in-hospital mortality and length of hospital stay in all patients with a diagnosis of DKA, including those with incomplete data, should be performed.**

**Answer;** The minimum sample size required to achieve our primary objectives is 387. The incomplete data were not having information on glucose level, in-hospital mortality and length of hospital stay. Further to this, the number of incomplete records were limited and would not affect the generalizability of the study as far as the required sample size is attained.
2. **The definition of DKA is questionable. The definition provided applies to ketosis, rather than to ketoacidosis. This is a relevant point: a mild ketosis, with hyperglycaemia and moderate ketonuria, has a different prognosis from that of a full ketoacidosis with reduced blood pH).**

**Answer:** The diagnosis and definition of DKA was made based of three important investigations including the pH level, level of consciousness of patients and bicarbonate
3. **The definition of ‘serum glucose rebound” is unclear (the amount of “increase in serum glucose level” needed to define the event is not specified), and this category seems to be altogether rather arbitrary. As a consequence, the definition of “poor glucose control” is weak. The analyses containing those two entities should be deleted.**

**Answer;**

Poor glycemic control was defined as the proportion of patients who were having plasma glucose >250 mg/dl after 6 hr therapy. Good Glycaemic control refers to the proportion of patients who were having plasma glucose ˂250 in 6 hr of therapy. A single increase in glucose level from the admission state was defined as poor Glycaemic control. If there was a trend of frequent increase in glucose level it indicates a rebound or fluctuation which is also part of poor Glycaemic control.
4. **There is no definition of “mild” or “severe” DKA**

**Answer;**

- **Mild DKA** is defined as (arterial pH, 7.25–7.30 and serum bicarbonate, 15–18 mEq/L)
- **Moderate DKA** is defined as (arterial pH, 7.00 to < 7.25 and serum bicarbonate, 10 to < 15 mEq/L)
- **Severe DKA** is defined as (arterial pH, < 7.00and serum bicarbonate, < 10 mEq/L).
  5. **There is no definition of comorbidity, nor of criteria used for the diagnosis of infection or omission as precipitating factors - all parameters analyzed as putative determinants of length of hospital stay.**

**Answer;** Comorbidity indicates the presence of other chronic conditions other than diabetes. the criteria for the reporting of infections as precipitating factors was based on the physicians note on the cause of the DKA for each incidence. Both data were available on patients’ record.
6. **I am quite surprised that blood glucose at admission (as a continuous variable) was not analyzed among putative determinants of length of hospital stay.**

**Answer;** We noticed that the number of times serum glucose fluctuates after admission was important factor for hospital stay instead of glucose level at admission. The fact that majority of patients were type one DM did not have an outlier of serum glucose to influence the hospital stay if there is no fluctuation the glucose concentration during hospital stay.
7. **The number of covariates in the multivariate analysis is too large. Considering the sample size and the number of events (about 80 cases of long stay), analyses with more than 7-8 covariates are not fully reliable. In addition, I do not understand the reason for which age was managed as a categorical variable with multiple categories (a procedure which would require large sample sizes) instead of a simpler management as a continuous variable. A good approach would be that of restricting multivariate analyses to those factors that are significantly associated with a long stay at univariate analyses, plus age, gender, admission glucose and admission ketones.**

**Answer;** We consider different factors as determinants of hospital stay. These factors were derived from literature. Therefore, we would like to include as many putative determinants as possible. In addition, we consider age as categorical variable as different age groups have different risk of hospital stay as evidenced by our analysis in which patients with age group of 35-44 had reduced hospital stay. Further, we also tried to highlight previously unrecognised factors that might affect the hospital stay in our study subjects.
8**. The main finding of this study is that the outcome (with respect to mortality and length of hospital stay) was more favourable than that reported in other African studies. The authors should discuss thoroughly this point, trying to identify determinants of this result (different case mix? Different definition of ketosis/ketoacidosis? Differences in availability of insulin supplies? Other differences in clinical settings or procedures?).**

**Answer;** We have discussed different reasons for the disparity in mortality including cost and availability of medications as compared to other African countries such as South Africa, Kenya, Tanzania and Gahana.
9. **With respect to determinants of hospital stay, the fact that infections require a longer stay is not surprising (the treatment of infection often requires a longer time). This should be clearly stated in the Discussion.**

**Answer;** The association between diagnosis of infection along with DKA and prolonged hospital stay was discussed in the discussion section along with other precipitating factors.
10. **The English language should be extensively revised.**

Answer; Edition of the English language have been taken place.
Reviewer #2:

1. **Multiple printing errors (including crucial definition e.g. LHS as‘dependent ‘factor)**

Answer; We have edited the definitions of the dependent variables.
2. **Multiple grammar errors; the format of the references need to be unified.**

**Answer,**

We go through each reference to address the uniformity of the reference styles and used Vancouver style throughout our citation.
3. **The description of background is redundant, and the discussion lacks in-depth insight of the topic**

**Answer;** We revised the background and discussion section in order to avoid redundant words and to increase the readability of the manuscript.
4. **There’s no clear definition for some key variables like severity of DKA, the rebound of ketosis.**

- **Poor glycemic control:** Proportion of patients having plasma glucose >250 mg/dl after 6 hr
- **Mild DKA** is defined as (arterial pH, 7.25–7.30 and serum bicarbonate, 15–18 mEq/L)
- **Moderate DKA** is defined as (arterial pH, 7.00 to < 7.25 and serum bicarbonate, 10 to < 15 mEq/L)
- **Severe DKA** is defined as (arterial pH, < 7.00and serum bicarbonate, < 10 mEq/L).
- **Rebound Ketonuria:** It is defined as the increase in ketone level once it is controlled with insulin management of fluid resuscitation.

5**. It’s a retrospective survey of the in-hospital treatment of adult DKA, it’s preferable that the protocol of DKA management in the very hospital be provided. In fact, what concerns me most is that there is some erroneous cognitions of DKA treatment. for example, the main stay of DKA treatment is not at all just the resolution of hyperglycemia. Instead, the treatment of ‘K’ &’A’ is more crucial than ‘D’(hyperglycemia ).Hence, the study revealed some treatment outcome of DKA in the hospital but could barely decide the determinants of DKA outcomes or achieve the goal of ‘improving the management of DKA.**

**Answer;** The treatment of DKA have been discussed in the methodology section how the hospital has been managing DKA. DKA treatment comprised of the resolution of dehydration, hyperglycaemia and metabolic acidosis which requires administration of insulin, fluid and bicarbonate. Data related to bicarbonate administration and fluid resuscitation are not fully recorded in the set-up and we kept this information under limitation of the study.
